# Knowledge and awareness of stroke and associated factors in the Saudi general population: a cross-sectional study

**DOI:** 10.3389/fneur.2023.1225980

**Published:** 2023-09-21

**Authors:** Reem Alzayer, Muna Barakat, Feras Jirjees, Aqeelah Alhamdan, Shatha Aloraifej, Sara Cherri, Sara Mansour, Sami El Khatib, Zelal Kharaba, Mohamad Rahal, Souheil Hallit, Diana Malaeb, Hassan Hosseini

**Affiliations:** ^1^Clinical Pharmacy Practice, Mohammed Al-Mana College for Medical Sciences (MACHS), Dammam, Saudi Arabia; ^2^Department of Clinical Pharmacy and Therapeutics, Faculty of Pharmacy, Applied Science Private University, Amman, Jordan; ^3^MEU Research Unit, Middle East University, Amman, Jordan; ^4^Clinical Pharmacy and Pharmacy Practice, University of Sharjah, Sharjah, United Arab Emirates; ^5^Almana General Hospital, Al Khobar, Saudi Arabia; ^6^School of Pharmacy, Lebanese International University, Beirut, Lebanon; ^7^Department of Biomedical Sciences, Lebanese International University, Bekaa, Lebanon; ^8^Center for Applied Mathematics and Bioinformatics (CAMB), Gulf University for Science and Technology, Mubarak Al-Abdullah, Kuwait; ^9^Department of Clinical Pharmacy, College of Pharmacy, Al Ain University, Abu Dhabi, United Arab Emirates; ^10^Faculty of Medical Sciences, Newcastle University, Newcastle upon Tyne, United Kingdom; ^11^School of Medicine and Medical Sciences, Holy Spirit University of Kaslik, Jounieh, Lebanon; ^12^Research Department, Psychiatric Hospital of the Cross, Jal El Dib, Lebanon; ^13^College of Pharmacy, Gulf Medical University, Ajman, United Arab Emirates; ^14^Neurology Department, Henri Mondor Hospital, AP-HP, Créteil, France; ^15^INSERM U955-E01, Institut Mondor de Recherche Biomedicale (IMRB), UPEC-Universite Paris-Est, Créteil, France

**Keywords:** stroke, awareness, knowledge, factors, Saudi Arabia

## Abstract

**Introduction:**

Stroke is a major cause of death and disability globally and in Saudi Arabia as well. Prevention and management of stroke depend highly on raising knowledge and awareness about the disease.

**Purpose:**

The purpose of this study was to evaluate Saudi adult's knowledge and awareness about stroke and determine the associated factors.

**Materials and methods:**

A cross-sectional online survey was conducted in May–July 2022 among Saudi citizens. Assessments of stroke knowledge about risk factors, symptoms, and response to stroke symptoms were evaluated. Logistic regression was conducted to assess the association between the socio-demographic characteristics and knowledge.

**Results:**

A total of 389 participants were enrolled with the majority (81.7%) being male participants. Less than half of the study subjects (43.3%) identified four out of five correct answers related to general knowledge about stroke. Almost all the participants were able to identify at least one risk factor associated with stroke. The majority of the participants (81.2%) believed that physical inactivity was the most common risk factor associated with stroke. Approximately three-quarters of participants considered difficulty speaking and understanding speech, followed by the sudden loss of consciousness as the most common stroke manifestation. Participants with a history of hypertension, dyslipidemia, and obesity had significantly higher odds of identifying at least one early stroke symptom (OR 2.271 [95% CI 1.402 3.677], 2.059 [95% CI 1.273 3.328], and 2.665 [95% CI 1.431 4.963], respectively).

**Conclusion:**

Our study revealed that participants have good knowledge about stroke. Nonetheless, further efforts are required to raise awareness and educate the public to optimize and ensure better treatment outcomes.

## 1. Introduction

Stroke is the third most common cause of disability and the second most common cause of death worldwide (1, 2). In addition to the significantly high mortality rate, stroke has a highly influential effect on morbidity causing up to 50% of long-term disabilities (2). As a result, stroke is a non-communicable disease that has significant economic and social repercussions that pose a great risk to public health (2). Thus, the public health burden of stroke is expected to increase over the coming decades due to population demographic and lifestyle changes, particularly in developing nations (2).

Saudi Arabia is a rapidly developing country and is the largest country in the Arabian Peninsula with over 32 million population out of which about 63% are native Saudis while the remaining are working-age immigrants, primarily from South and East Asia (3, 4). In the past two decades, environmental and lifestyle factors have changed in Saudi Arabia increasing the risk and incidence of stroke (5). In 2012, stroke resulted in more than 14,000 mortalities in Saudi Arabia leading it to be the second common cause of death (3, 4). Moreover, the expectation is that the incidence of stroke and the fatality rate will increase approximately 2-fold by 2030 in the country (3).

The high prevalence of stroke is partially attributable to a lack of community awareness about stroke risk factors (2). Stroke risk factors include lifestyle aspects such as unhealthy diet, smoking, hypertension, diabetes mellitus, dyslipidemia, and cardiovascular diseases (6). Studies from industrialized and developing nations demonstrate that respondents typically have suboptimal stroke knowledge and recognition rates for any identified stroke risk factors and management (2, 7–10). In addition, recognizing early warning symptoms of stroke is crucial to implement accurate timely measurement and ensure better treatment outcomes (11). Rapid thrombolysis therapy during the first 4–6 h from stroke clinical signs onset lowers the incidence of disabilities and enhances clinical outcomes (11). Literature reported that failure to recognize stroke early symptoms is a significant contributing cause of delays in medical reporting of stroke or late arrival at the emergency department causing permanent physical and mental disabilities and deaths (2, 3). Therefore, increased awareness and knowledge about the clinical signs and management among the public could minimize the timeframe between the onset of symptoms and the fast delivery of thrombolytic drugs which improves patient outcomes (11). There has not been a comprehensive study done in Saudi Arabia to evaluate the level of public knowledge about stroke-related risk factors, symptoms, and consequences. Therefore, this study aimed to evaluate the Saudi adult population's general knowledge and awareness about stroke and determine factors associated with their knowledge.

## 2. Materials and methods

### 2.1. Study design and population

This cross-sectional study consists of an online survey conducted between May and July 2022 among Saudi citizens from all regions of the country. Eligibility criteria were (i) being 18 years or older and (ii) having Saudi nationality and currently residing in the country. Participants with a history of stroke and those who were unable to fill out the survey independently were excluded.

The online survey was designed using Google Forms and was disseminated through different social media platforms (LinkedIn, WhatsApp, and Telegram). People were invited to participate and to share the survey with others which allowed for snowball sampling. Participants completed the survey without the assistance of researchers to avoid any potential bias in their responses.

### 2.2. Minimal sample size

Using Raosoft software, the minimal target sample size was approximately 385 individuals based on another study and taking a confidence interval of 95%, a standard deviation of 0.5, and a margin of error of 5% (12).

### 2.3. Ethics approval

The Institutional Review Board (IRB) approval was obtained from the Scientific Research Unit at Almana College for Medical Sciences, Dammam, Saudi Arabia (Approval reference # SR/RP/82). The study followed the ethical standards of the Declaration of Helsinki. Participation was completely voluntary, and responses were anonymous. Before accessing the survey, all participants gave written informed consent (online consent) to take part in the study.

### 2.4. Instrument

The online survey collected data on stroke, such as general knowledge, risk factors, early symptoms, and consequences, as well as demographic and socioeconomic factors. Pilot testing was performed on 35 individuals, and the data collected were not included in the final analysis. The survey was self-administered by participants and was available in Arabic, the native language of Saudi Arabia. A written Participant Information Statement (PIS) describing the main purpose of the study and the expected time required to complete the questionnaire (10–15 min) was provided to participants at the start of the survey.

The survey was developed based on previous literature that used a set of questions to accurately capture multiple aspects of stroke awareness and general knowledge in different populations (13–17). The survey questions were mostly adapted from a previous study conducted in Jordan with a similar aim of assessing stroke awareness and general knowledge among the general population (10). The first section of the survey covered the demographic and socioeconomic factors such as age (< 30, 30–49, 50–70, and >70 years), smoking status, marital status, employment status, family income, residence area (urban, rural, Al Badeya), level of education, and past medical history (preexisting chronic disease as reported by participants). Monthly income was categorized into four groups: <5,000 SR, 5,000–10,000 SR, >10,000–≤20,000 SR, and >20,000 SR, with 1 US Dollars equals 3.75 SR (18).

The second section of the survey assessed general stroke knowledge. Participants responded to whether stroke is a disease that (1) affects the brain, (2) is common in older people, (3) is a contagious disease, (4) is a hereditary disease, and (5) is preventable. This section also examined participants' knowledge of stroke risk factors such as hypertension, smoking, alcohol intake, dyslipidemia, diabetes, physical inactivity, cardiovascular disease, obesity, old age, and emotional stress. In addition, knowledge of early warning signs was evaluated, and response options included the following: (1) numbness of one side of the body such as the face or arms, (2) difficulty speaking or understanding, (3) visual impairment/double vision, (4) loss of coordination, (5) severe headache with no known cause, (6) sudden dizziness, and (7) loss of consciousness. Participants were also asked to identify the consequences of stroke which included the following: (1) memory problems (i.e., inability to speak and remember or understand speech), (2) movement problems (i.e., balance problems and one-sided paralysis), (3) visual problems (i.e., loss or blurred vision), (4) emotional and personality changes (i.e., depression and mood changes), and (5) long-term disabilities. In the last section, the survey looked at the willingness of the participants to take a patient to hospital if symptoms of stroke were observed. In a previous study conducted in Jordan (10), participants were granted one point for each correct answer to the above questions; however, there was no identifiable cutoff point for a satisfactory level of stroke knowledge. Similarly, in our study, we computed the number of correctly identified risk factors, early symptoms, and consequences by giving one point for each correct answer to the aforementioned questions. Correct answers for each question were then computed, and a good level of knowledge was considered to be >50%.

### 2.5. Statistical analysis

Descriptive statistics were used to describe socio-demographic characteristics and assess stroke general knowledge, risk factors, early symptoms, and consequences that were identified by participants. All continuous variables were presented as mean and standard deviation (SD), while categorical variables were presented as frequencies (n) and percentages (%). The associations of stroke risk factors, early symptoms, and response to a patient experiencing a stroke with the socio-demographic characteristics and past medical history were examined using Pearson's chi-square or Fisher's exact test if applicable. A logistic regression model was then conducted to assess the relationship between the variables that showed *p*-value < 0.2 in the bivariate analysis and identified stroke risk factors, early symptoms, and consequences. *P*-values < 0.05 were considered statistically significant. Statistical analysis was performed using the Statistical Package for Social Sciences version (SPSS) 25.0.

## 3. Results

### 3.1. Sample description

A total of 873 Saudi adults participated in this study, of which 389 participants who completed all sections on the variables of interest were included. Of these, 318 (81.7%) were male participants, 149 (61.7%) were between 30 and 49 years of age, and more than half were married (67.9%). Most participants (63.2%) were residing in urban areas with 42.9% of them living in the capital of Saudi Arabia. The majority of the participants had primary school level (40.1%) and part-time jobs (49.6%). The most common concomitant disease was dyslipidemia (51.2%), followed by hypertension (44.7%) and arrhythmia (41.9%). Almost half of the participants heard of a stroke (54.7%). The socio-demographic characteristics of participants are shown in Table 1.

**Table 1:** Participants' socio-demographic characteristics, past medical history, and familiarity with stroke.

**Variables (*****N*** = **389)**		**Frequency (%)**
**Socio-demographic characteristics**		
Sex	Male	318 (81.7)
	Female	71 (18)
Age (years)	< 30	54 (13.9)
	30–49	240 (61.7)
	50–70	92 (23.7)
	>70	3 (0.8)
Residence area	Urban	246 (63.2)
	Rural	124 (31.9)
	Al Badeya	19 (4.9)
Residency	Al Riyad Capital	167 (42.9)
	West area	4 (1)
	East area	30 (7.7)
	South area	40 (10.3)
	North area	148 (38)
Marital status	Single	37 (9.5)
	Married	264 (67.9)
	Divorced	84 (21.6)
	Widowed	4 (1)
Education (years)	Uneducated	23 (5.9)
	Primary level	156 (40.1)
	Middle level	126 (32.4)
	High school level	56 (14.4)
	University	28 (7.2)
Employment status	Student	35 (9)
	Unemployed	94 (24.2)
	Employed with part-time job	193 (49.6)
	Employed with full-time job	64 (16.5)
	Businessman	3 (0.8)
Work field	Healthcare field	322 (82.8)
	Non-healthcare filed	67 (17.2)
Income level (Riyal Saudi)	Less than 5,000	205 (52.7)
	Between 5,000 and 10,000	134 (34.4)
	More than 10,000 and less than 20,000	42 (10.8)
	More than 20,000	8 (2.1)
History of smoking	Yes	320 (82.3)
Past medical history	Hypertension	215 (44.7)
	Diabetes Mellitus	99 (25.4)
	Dyslipidemia	199 (51.2)
	Arrhythmia	163 (41.9)
	Obesity	97 (24.9)
Familiarity with stroke	History of stroke in the family	206 (53)
	Personally know someone with stroke	298 (76.6)

**Figure 1:**
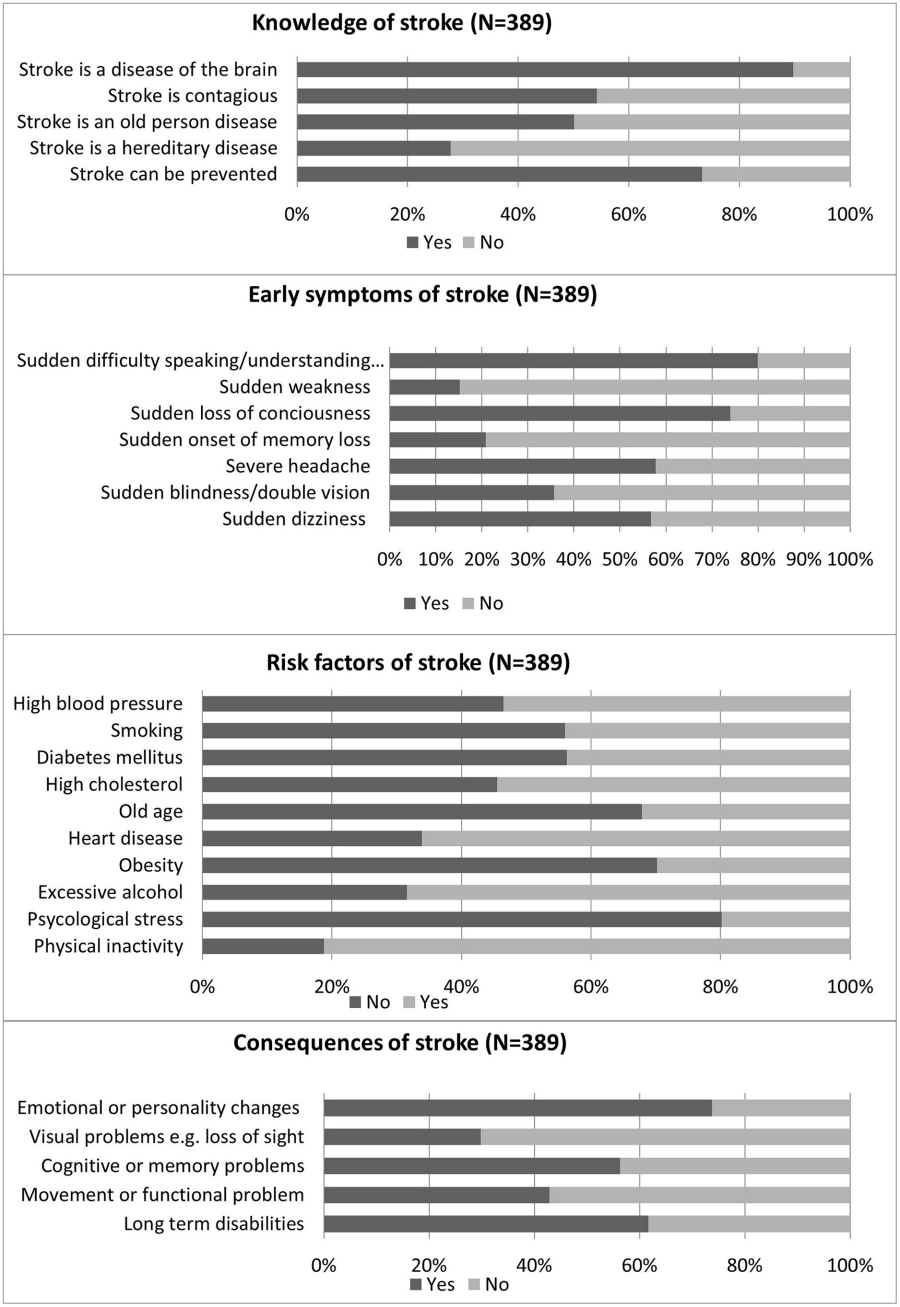
Knowledge among the participants.

The sample showed a variable level of knowledge about stroke (Figure 1, Table 2). The majority were aware that stroke is a brain disease that is preventable and not contagious (89.7%, 73.3%, and 54.2%, respectively). Less than half of the participants (43.3%) could identify four out of five correct answers related to general knowledge about stroke. Furthermore, most of the participants (81.2%) believed that physical inactivity was the most common risk factor for stroke, followed by excessive alcohol (68.4%) and heart disease (66.1%). The most common warning signs were “sudden difficulty in speaking or understanding speech” and “sudden loss of consciousness” (79.9% and 74.0%, respectively). Only 1.5% of the participants recognized all the risk factors and identified all the symptoms, and 1.8% stated all possible consequences of stroke.

**Table 2:** Number of stroke risk factors, early symptoms, and consequences that were identified by the participants.

**Variables (*N* = 389)**		**Frequency (%)**	**Cumulative, frequency (%)**
Number of correct answers regarding stroke in the general knowledge	Zero	2 (0.5)	2 (0.5)
	One	7 (1.8)	9 (2.3)
	Two	105 (27)	114 (29.3)
	Three	95 (24.4)	209 (53.7)
	Four	169 (43.4)	378 (97.2)
	Five	11 (2.8)	389 (100)
Number of identified risk factors of stroke	Zero	2 (0.5)	2 (0.5)
	One	1 (0.3)	3(0.8)
	Two	2 (0.5)	5 (1.3)
	Three	15 (3.9)	20 (5.1)
	Four	82 (21.1)	102 (26.2)
	Five	216 (55.5)	318 (81.7)
	Six	60 (15.4)	378 (97.2)
	Seven	2 (0.5)	380 (97.7)
	Eight	1 (0.3)	381 (97.9)
	Nine	2 (0.5)	383 (98.5)
	Ten	6 (1.5)	389 (100)
Number of identified early symptoms of stroke	Zero	2 (0.5)	2 (0.5)
	One	2 (0.5)	4 (1)
	Two	74 (19)	78 (20.1)
	Three	136 (35)	214 (55)
	Four	125 (32.1)	339 (87.1)
	Five	40 (10.3)	379 (97.4)
	Six	4 (1)	383 (98.5)
	Seven	6 (1.5)	389 (100)
Number of identified consequences of stroke	One	13 (3.3)	13 (3.3)
	Two	142 (36.5)	155 (39.8)
	Three	211 (54.2)	366 (94.1)
	Four	16 (4.1)	382 (98.2)
	Five	7 (1.8)	389 (100)

Almost half of the participants stated that the source of information about stroke was the healthcare team and Internet/social media with 55.4% and 55.2%, respectively. In addition, more than half of the participants (57.5%) relied on TV and radio for their information.

### 3.2. Bivariate analysis

A significantly higher proportion of male participants vs. female participants (100% vs. 97.2%) and those working in healthcare vs. non-healthcare were able to correctly identify at least one stroke risk factor. Moreover, a significantly higher proportion of participants who lived in the east area (73.3%) followed by north area (65.5%) vs. other regions in Saudi Arabia and those who were single and divorced (64.9% and 100%, respectively) vs. married and widowed (53.8% and 34.5%, respectively) recognized at least one warning symptom of stroke. A significantly higher proportion of student participants and unemployed (74.3% and 71.3%, respectively) compared to those employed part-time or full-time job and businessmen/women (48.2%, 51.6%, and 66.7%, respectively) and those who had higher income level correctly identified the early warning symptoms of stroke (Table 3). In addition, a significantly higher proportion of participants who identified at least one correct early symptom of stroke had a history of hypertension, dyslipidemia, and kidney disease (69.3%, 70.9%, and 63.6%) (Table 3). Concerning the response to taking a patient to the hospital with stroke symptoms, a significantly higher number of correct responses were related to having a history of dyslipidemia (50.8%) vs. with no history of dyslipidemia (38.9%) (Table 4).

**Table 3:** Association of risk factors, early symptoms, and consequences of stroke with the socio-demographic characteristics and past medical history.

**Variables (*****N*** = **389)**		**Risk factor(s) identified (**≥**1)**			**Early symptom(s) identified (**≥**1)**		
		**Yes (*****n** =* **387), n (%)**	**No (*****n** =* **2), n (%)**	***P*** **-value**	**Yes (*****n** =* **221), n (%)**	**No (*****n** =* **168), n (%)**	***P*** **-value**
**Socio-demographic characteristics**							
**Sex**	Male	318 (100)	0 (0)	**0.03**	178 (56)	140 (44)	0.51
	Female	69 (97.2)	2 (2.8)		43 (60.6)	28 (39.4)	
Age (years)	< 30	54 (100)	0 (0)	1.00	34 (63)	20 (37)	0.17
	30–49	238 (99.2)	2 (0.8)		138 (57.5)	102 (42.5)	
	50–70	92 (100)	0 (0)		49 (53.3)	43 (46.7)	
	>70	3 (100)	0 (0)		0 (0)	3 (100)	
Residence area	Urban	245 (99.6)	1 (0.4)	0.26	141 (57.3)	105 (42.7)	0.41
	Rural	124 (100)	0 (0)		72 (58.1)	52 (41.9)	
	Al Badeya	18 (94.7)	1 (5.3)		8 (42.1)	11 (57.9)	
Residency	Al Riyad capital	166 (99.4)	1 (0.6)	0.09	83 (49.7)	84 (50.3)	**0.01**
	West area	4 (100)	0 (0)		1 (25)	3 (75)	
	East area	29 (96.7)	1 (3.3)		22 (73.3)	8 (26.7)	
	South area	40 (100)	0 (0)		18 (45)	22 (55)	
	North area	148 (100)	0 (0)		97 (65.5)	51 (34.5)	
Marital status	Single	37 (100)	0 (0)	1.00	24 (64.9)	13 (35.1)	**0.01**
	Married	262 (99.2)	2 (0.8)		142 (53.8)	122 (46.2)	
	Divorced	4 (100)	0 (0)		4 (100)	0 (0)	
	Widowed	84 (100)	0 (0)		29 (34.5)	55 (65.5)	
Educational level	Uneducated	23 (100)	0 (0)	0.01	12 (52.2)	11 (47.8)	0.16
	Primary level	156 (100)	0 (0)		82 (52.6)	74 (47.4)	
	Middle level	126 (100)	0 (0)		79 (62.7)	47 (37.3)	
	High school	56 (100)	0 (0)		36 (64.3)	20 (35.7)	
	University	26 (92.9)	2 (7.1)		12 (42.9)	16 (57.1)	
Employment status	Student	35 (100)	0 (0)	**0.05**	26 (74.3)	9 (25.7)	**0.01**
	Unemployed	94 (100)	0 (0)		67 (71.3)	27 (28.7)	
	Employed with part-time	193 (100)	0 (0)		93 (48.2)	100 (51.8)	
	Employed with full-time	62 (96.9)	2 (3.1)		33 (51.6)	31 (48.4)	
	Businessman	3 (100)	0 (0)		2 (66.7)	1 (33.3)	
Work field	Health care field	322 (100)	0 (0)	**0.03**	179 (55.6)	143 (44.4)	0.34
	Non-healthcare filed	65 (97)	2 (3)		42 (62.7)	25 (37.3)	
Income level	Less than 5,000 Riyal Saudi	205 (100)	0 (0)	0.13	93 (45.4)	112 (54.6)	**0.01**
	Between 5,000 and 10,000	133 (99.3)	1 (0.7)		88 (65.7)	46 (34.3)	
	More than 10,000 and less than 20,000	41 (97.6)	1 (2.4)		32 (76.2)	10 (23.8)	
	More than 20,000	8 (100)	0 (0)		8 (100)	0 (0)	
History of smoking (≥1 year)	No	68 (98.6)	1 (1.4)	0.32	45 (65.2)	24 (34.8)	0.14
	Yes	319 (99.7)	1 (0.3)		176 (55)	144 (45)	
**Past medical history**							
Hypertension	No	172 (98.9)	2 (1.1)	0.19	72 (41.4)	102 (58.6)	**0.01**
	Yes	215 (100)	0 (0)		149 (69.3)	66 (30.7)	
Diabetes mellitus	No	288 (99.3)	2 (0.7)	1.00	167 (57.6)	123 (42.4)	0.64
	Yes	99 (100)	0 (0)		54 (54.5)	45 (45.5)	
Dyslipidemia	No	188 (98.9)	2 (1.1)	0.24	80 (42.1)	110 (57.9)	**0.01**
	Yes	199 (100)	0 (0)		141 (70.9)	58 (29.1)	
Arrhythmia	No	224 (99.1)	2 (0.9)	0.51	124 (54.9)	102 (45.1)	0.41
	Yes	163 (100)	0 (0)		97 (59.5)	66 (40.5)	
Kidney disease	No	192 (99)	2 (1)	0.25	97 (50)	97 (50)	**0.01**
	Yes	195 (100)	0 (0)		124 (63.6)	71 (36.4)	
Peptic ulcer	No	211 (99.1)	2 (0.9)	0.50	116 (54.5)	97 (45.5)	0.31
	Yes	176 (100)	0 (0)		105 (59.7)	71 (40.3)	
Depression	No	144 (98.6)	2 (1.4)	0.14	75 (51.4)	71 (48.6)	0.11
	Yes	243 (100)	0 (0)		146 (60.1)	97 (39.9)	
Obesity	No	290 (99.3)	2 (0.7)	1.00	142 (48.6)	150 (51.4)	**0.01**
	Yes	97 (100)	0 (0)		79 (81.4)	18 (18.6)	

Fisher's exact test was used when the cell counts were < 5.

Numbers in bold indicate significant *p*-values.

**Table 4:** Association of taking a patient who is experiencing stroke to the hospital with socio -demographic characteristics and past medical history.

**Variables (*****N*** = **389)**		**Taking a patient who is experiencing stroke to the hospital**		
		**Yes (*****n** =* **175)**, ***n*** **(%)**	**No (*****n** =* **214)**, ***n*** **(%)**	***P*** **-value**
**Socio-demographic characteristics**				
Sex	Male	144 (45.3)	174 (54.7)	0.89
	Female	31 (43.7)	40 (56.3)	
Age (years)	< 30	26 (48.1)	28 (51.9)	0.46
	30–49	113 (47.1)	127 (52.9)	
	50–70	35 (38)	57 (62)	
	>70	1 (33.3)	2 (66.7)	
Residence area	Urban	120 (48.8)	126 (51.2)	0.15
	Rural	48 (38.7)	76 (61.3)	
	Al Badeya	7 (36.8)	12 (63.2)	
Residency	Al Riyad capital	71 (42.5)	96 (57.5)	0.79
	West area	2 (50)	2 (50)	
	East area	15 (50)	15 (50)	
	South area	16 (40)	24 (60)	
	North area	71 (48)	77 (52)	
Marital status	Single	117 (44.3)	147 (55.7)	0.26
	Married	22 (59.5)	147 (55.7)	
	Divorced	2 (50)	2 (50)	
	Widowed	34 (40.5)	50 (59.5)	
Educational level	Uneducated	13 (56.5)	10 (43.5)	0.40
	Primary level	72 (46.2)	84 (53.8)	
	Middle level	49 (38.9)	77 (61.1)	
	High school	26 (46.4)	30 (53.6)	
	University	15 (53.6)	13 (46.4)	
Employment status	Student	14 (40)	21 (60)	0.93
	Unemployed	43 (45.7)	51 (54.3)	
	Employed with part-time job	90 (46.6)	103 (53.4)	
	Employed with full-time job	27 (42.2)	37 (57.8)	
	Businessman	1 (33.3)	2 (66.7)	
Work field	Healthcare	148 (46)	174 (54)	0.42
	Non-healthcare	27 (40.3)	40 (59.7)	
Income level	< 5,000 Riyal Saudi	86 (42)	119 (58)	0.39
	Between 5,000 and 10,000 Riyal Saudi	68 (50.7)	66 (49.3)	
	More than 10,000 and < 20,000 Riyal Saudi	17 (40.5)	25 (59.5)	
	More than 20,000 Riyal Saudi	4 (50)	4 (50)	
History of smoking (≥1 year)	No	37 (53.6)	32 (46.4)	0.14
	Yes	138 (43.1)	182 (56.9)	
**Past medical history**				
Hypertension	No	80 (46)	94 (54)	0.76
	Yes	95 (44.2)	120 (55.8)	
Diabetes mellitus	No	138 (47.6)	152 (52.4)	0.08
	Yes	37 (37.4)	62 (62.6)	
Dyslipidemia	No	74 (38.9)	116 (61.1)	**0.03**
	Yes	101 (50.8)	98 (49.2)
Arrhythmia	No	99 (43.8)	127 (56.2)	0.60
	Yes	76 (46.6)	87 (53.4)	
Kidney disease	No	82 (42.3)	112 (57.7)	0.31
	Yes	93 (47.7)	102 (52.3)	
Peptic ulcer	No	102 (47.9)	111 (52.1)	0.22
	Yes	73 (41.5)	103 (58.5)	
Depression	No	71 (48.6)	75 (51.4)	0.29
	Yes	104 (42.8)	139 (57.2)	
Obesity	No	123 (42.1)	169 (57.9)	0.06
	Yes	52 (53.6)	45 (46.4)	

Fisher's exact test was used when the cell counts were < 5.

Numbers in bold indicate significant *p*-values.

### 3.3. Multivariable analysis

When taking the identification of at least one early symptom of stroke as the dependent variable, participants with a history of hypertension, dyslipidemia, and obesity vs. those with no history had significantly higher odds (OR of 2.271 [95% CI 1.402 3.677], 2.059 [95% CI 1.273 3.328], and 2.665 [95% CI 1.431 4.963], respectively) (Table 5).

**Table 5:** Multivariate analysis.

**Variables (*N* = 389)**	**β (SE)**	**OR (95% CI)**	***P*-value**
**Risk factor(s) identified (**≥**1)**			
Sex (female vs. male^*^)	−17.375 (1891.9)	0	0.99
Work field (non-healthcare field vs. healthcare field^*^)	−17.441 (1886.842)	0	0.99
**Early symptom(s) identified (**≥**1)**			
**Marital status**			
Single vs. married	−0.392 (0.297)	0.675 (0.378;1.208)	0.19
Widowed vs. married	0.157 (0.464)	1.17 (0.471;2.907)	0.74
Divorced vs. married	−21.944 (19857.6)	0	0.99
**Employment status**			
Unemployed vs. student	0.965 (1.557)	2.625 (0.124;55.474)	0.54
Employed with part-time job vs. student	0.560 (1.504)	1.75 (0.092;33.36)	0.71
Employed with full-time job vs. student	−0.159 (1.493)	0.853 (0.046;15.912)	0.92
Businessman vs. student	0.018 (1.512)	1.018 (0.053;19.72)	0.99
**Income level (Riyal Saudi)**			
Between 5,000 and 10,000 vs. < 5,000	0.438 (0.269)	1.55 (0.915;2.627)	0.10
More than 10,000 and < 20,000 vs. < 5,000	0.599 (0.445)	1.82 (0.761;4.353)	0.18
More than 20,000 vs. < 5,000	20.148 (13800.07)	562781716.4 (0)	0.99
History of hypertension (yes vs. no^*^)	0.820 (0.246)	2.271 (1.402; 3.677)	**0.01**
History of dyslipidemia (yes vs. no^*^)	0.722 (0.245)	2.059 (1.273; 3.328)	**0.03**
History of kidney disease (yes vs. no^*^)	0.405 (0.238)	1.499 (0.94; 2.388)	0.89
History of obesity (yes vs. no^*^)	0.980 (0.317)	2.665 (1.431; 4.963)	**0.01**

β, Beta; SE, standard error; OR, adjusted ratio; CI, confidence interval.

Logistic regression taking the identification of stroke risk factors and stroke early symptoms as the dependent variables and socio-demographic factors (gender, residence area, educational level, work field, employment status, marital status, income level, history of hypertension, history of dyslipidemia, history of kidney disease, and history of obesity) as independent variables.

Bold values represent significant values.

* Represent reference.

## 4. Discussion

In previous studies, the Saudi population has been shown to have inadequate health literacy associated with poor health information knowledge (19, 20). So, this study assessed factors related to the general Saudi population's knowledge about stroke, its risk factors, early symptoms, and consequences. However, the results showed that the majority of the participants were aware that stroke is related to the brain and recognized that it is not contagious. On the other hand, a very low percentage of participants recognized all the risk factors and were able to identify all the symptoms and possible consequences of a stroke. This is consistent with an integrative review that found that general knowledge about recognizing stroke is poor even among hospitalized patients (21, 22). In addition, a systematic review showed that stroke knowledge among different populations was suboptimal (23). A similar conclusion was found in a review summarizing the findings of 15 studies where it was also found that the level of knowledge about stroke is generally low (24).

In this study, almost all participants identified at least one correct risk factor and consequence of stroke. This is higher than what was reported in similar studies assessing the populations' knowledge of stroke potential risk factors and warning symptoms in the USA (25, 26), Australia (27), Norway (28), Portugal (29), Spain (30), Korea (31), and Oman (32). However, almost all participants in two recent studies conducted in Lebanon (33) and Jordan (10) were able to identify at least one correct consequence of stroke, consistent with our findings. Our findings can be interpreted by the fact that the majority of our participants were workers in the medical field and there is advancement in health services, combined with other factors such as improved and more accessible public education, better life conditions, and greater health funding, all of which contribute to increased health awareness among the community (34).

Our findings also showed that more than 80% of participants identified physical inactivity as the most common risk factor for stroke, followed by excessive alcohol intake and heart disease. However, previous studies conducted in Saudi Arabia found that more than 75% of participants identified hypertension as the most common cause of stroke (5, 35). Hypertension and psychological stress were identified as the most common risk factors for stroke by the participants of similar studies conducted in Lebanon (13, 36) and Morocco (37). Hypertension was also reported as a common risk factor for stroke by participants in the USA, along with smoking, physical inactivity, and diabetes (26). Hypertension was also frequently reported as a common risk factor by participants in Spain in addition to alcohol intake and smoking (30). In Oman, participants also recognized hypertension as the most common risk factor for stroke (32). Although hypertension is an important risk factor for stroke, the participants in this study underappreciated it. A similar observation was also found in a study conducted in Australia (27). It was also noted that diabetes, which is another important risk factor for stroke (37), was also underappreciated by the participants of this study and other similar studies conducted in Lebanon (33), Korea (31), Australia (27), Norway (28), and Ireland (38).

Approximately three-quarters of this study's participants considered dysarthria, which is difficulty speaking or understanding speech, as the most common sign of stroke followed by sudden loss of consciousness. Similar studies in Jordan (10), Lebanon (33), Australia (27), and Ireland (38) reported dysarthria as the most common sign of stroke. On the other hand, participants of studies conducted in Oman (32), Korea (31), Spain (30), and Nigeria (39) reported weakness or paralysis of one side of the body as the most common sign of stroke. Numbness, weakness, and speech difficulties were reported as the most common symptoms of stroke in studies conducted in Norway (28) and in the United States (25, 26), while, in Australia, participants reported blurred and double vision or loss of vision as the most common early stroke symptoms (27).

Surprisingly, less than half the participants reported that they would take a patient suffering a stroke to the hospital immediately. This is in contrary to the results of a study conducted in 2011 in Saudi Arabia, where approximately 87% of participants recognized the need for urgent medical attention (40). However, the aforementioned study was not restricted to Saudi citizens and included mainly younger participants (aged between 20 and 30 years old) with higher educational attainments compared to our study. Another study conducted in 2017 found that older Saudi citizens also failed to recognize the importance of calling the emergency or taking a patient suffering from a stroke to a hospital as they said that they would advise the patient to take rest to recover from a stroke (41). However, the percentage of participants in this study who were aware of the importance of seeking medical help for a patient suffering from a stroke is consistent with what was reported in a systematic review of international studies (23). In other countries, including Oman, Lebanon, Jordan, and Spain, most of the participants were aware of the importance of going to a hospital emergency as early as possible after a stroke is identified (10, 30, 32, 33).

In this study, participants with higher educational levels, those with full-time job, and those working in the medical field had better knowledge of stroke risk factors and early symptoms. Higher education and employment were found to play an important role in participants' knowledge and awareness regarding stroke in previous studies conducted in Saudi Arabia (40, 41), Lebanon (33), Jordan (10), Oman (32), USA (25), Ireland (38), Norway (28), Spain (30), and Portugal (29). This is expected as higher education is usually accompanied by better opportunities for employment and thus better income and better access to health services and health insurance, which help individuals in accessing and understanding health information. It was also noted that the participants who suffer from obesity, diabetes, and dyslipidemia were more aware of the importance of seeking medical help for a patient suffering from a stroke. Generally, people with chronic diseases are more likely to have better evaluations of health issues as they visit healthcare providers more often and thus get somewhat exposed to health-related information more often compared to healthy individuals (42, 43). In addition, people with chronic conditions tend to use the Internet more often to retrieve health-related information (44).

This study has several limitations. The results could not be representative of the entire Saudi population as the majority were men and worked in the medical field. Additionally, cross-sectional design cannot infer causality. Information bias could also exist as the study questionnaire was online and answers were self-reported. Selection bias might have also occurred since the sample was not randomly selected but rather gathered using the snowball sampling technique. Residual confounding bias is also possible since there might be factors related to stroke awareness that were not measured in this study. Furthermore, we did not validate the knowledge scale, and we assessed only the level of awareness regarding stroke. In addition, our analysis regarding the identification of one symptom/risk factor for stroke was based on other studies (13–17). On the other hand, our study has several strengths as it added data to the literature about stroke disease awareness which highlighted the lack of knowledge regarding stroke among the Saudi population.

## 5. Conclusion

In general, the Saudi population has high levels of health literacy regarding stroke risk factors, symptoms, and consequences. Our study pinpoints that more awareness campaigns for under-educated, unemployed participants, as well as for those living in suburban areas are mandated to raise awareness about stroke. A better understanding of many elements of the stroke was related to higher educational attainment, living in urban areas, and having a job. To support our findings, additional research with a larger sample size and a more representative sample of the Saudi population is required.

## Data availability statement

The raw data supporting the conclusions of this article will be made available by the authors, without undue reservation.

## Ethics statement

The studies involving human participants were reviewed and approved by the Scientific Research Unit at Almana College for Medical Sciences, Dammam, Saudi Arabia (Approval reference # SR/RP/82). The patients/participants provided their written informed consent to participate in this study.

## Author contributions

DM and HH: conceptualization. DM and RA: investigation. DM, SH, and RA: methodology. RA: project administration. DM and SH: supervision. RA, MB, FJ, AA, and ZK: writing—original draft preparation. SC, SM, SE, and MR: writing—reviewing and editing. All authors read and approved the final version of the manuscript.
